# Investigating trends in asthma and COPD through multiple data sources: A small area study

**DOI:** 10.1016/j.sste.2016.05.004

**Published:** 2016-11

**Authors:** Areti Boulieri, Anna Hansell, Marta Blangiardo

**Affiliations:** a Department of Epidemiology and Biostatistics, MRC-PHE Centre for Environment and Health, Imperial College London, London, UK; b UK Small Area Health Statistics Unit, MRC-PHE Centre for Environment and Health, Imperial College London, London, UK; c Imperial College Healthcare NHS Trust, London, UK

**Keywords:** Space-time analysis, Detection, Asthma and COPD

## Abstract

This paper investigates trends in asthma and COPD by using multiple data sources to help understanding the relationships between disease prevalence, morbidity and mortality. GP drug prescriptions, hospital admissions, and deaths are analysed at clinical commissioning group (CCG) level in England from August 2010 to March 2011. A Bayesian hierarchical model is used for the analysis, which takes into account the complex space and time dependencies of asthma and COPD, while it is also able to detect unusual areas. Main findings show important discrepancies across the different data sources, reflecting the different groups of patients that are represented. In addition, the detection mechanism that is provided by the model, together with inference on the spatial, and temporal variation, provide a better picture of the respiratory health problem.

## Highlights

• Bayesian modelling of asthma and COPD.
• Use of multiple data sources to assess disease prevalence, morbidity and mortality.
• Spatial and temporal patterns across England over the period August 2010 to March 2011.
• Detection of areas with unusual temporal patterns.

## 1 Introduction

Asthma and chronic obstructive pulmonary disease (COPD) are the most common chronic respiratory conditions worldwide, contributing to heavy social and economic burden (World Health Organization, 2012).

The number of people suffering from asthma in 2014 was estimated to be 334 million around the world (World Health Organization, 2014) and this number is projected to rise to 400 million by 2025. Around 250,000 deaths per year are caused by the disease, with the majority of them considered to be preventable (Masoli et al., 2004). COPD has a lower prevalence of 64 million people but much higher mortality, with 3 million deaths annually, an estimated 6% of all deaths worldwide. COPD is predicted to become the third leading cause of death by 2030 (World Health Organization, 2014). In the UK, asthma affects 1 in 5 households, and COPD is the fifth leading cause of death after cancer and cardiovascular disease (Masoli et al., 2004).

Asthma and COPD have similarities in symptoms and treatment and there may be considerable overlap between these conditions making them difficult to distinguish clinically (Drazen et al., 2015). Asthma commonly starts in childhood and is often allergic in origin, while a large proportion of COPD is caused by smoking and the condition starts in mid to later life. Common symptoms in both conditions are shortness of breath and wheeze, with worsening of symptoms with respiratory infections, with similarities in treatments of bronchodilators, steroids and antibiotics for infections. Triggers for exacerbations, the major determinants of admissions and possibly deaths are likely to be influenced by infectious disease trends and by common environmental factors with a spatio-temporal structure, such as air pollution (Eeftens et al., 2012).

A study of trends of these chronic respiratory diseases is important, as it allows a better understanding of the characteristics of the disease, to determine whether health policies or preventive measures are effective, and to identify high-risk populations that might require additional care and treatments. A challenge, however, is the choice of data to use for asthma and COPD analyses. Studies on mortality (Sin et al., 2006) investigate only the highest degree of severity and findings may differ from those for hospital admissions particularly for asthma (Hansell et al., 2003), which is a more heterogeneous condition. Most studies have used data from secondary and tertiary care, such as hospital admissions and emergency care data, but these will not capture milder cases seen in primary care. It is estimated that only 20% of asthmatic patients and less than half of COPD patients suffer from severe symptoms (Lindebeg et al., 2006).

A useful addition to asthma and COPD research is the use of General Practice (GP) drug prescription data which consist in the numbers of items that are prescribed in England by GPs and are dispensed anywhere in UK or Europe. GP drug prescriptions can be very relevant for asthma and COPD as these are long-term conditions that are controlled by regular medication. These capture patients of any severity of the disease, from mild to severe, and hence they can provide a general picture of the respiratory health of the population at small area level. Only a few authors have used GP drug prescriptions to investigate asthma and COPD trends (Hansell, Hollowell, McNiece, Nichols, Strachan, 2003, Laurent, Pedrono, Filleul, Segala, Lefranc, Schillinger, Rivière, Bard, 2009, Naureckas, Dukic, Bao, Rathouz, 2005, Sofianopoulou, Rushton, Diggle, Pless-Mulloli, 2013, Vegni, Castelli, Auxilia, Wilkinson, 2005).

In addition, the geographical trends of asthma and COPD have only been studied by a few authors. Hansell et al. (2003) found COPD mortality, hospital admissions and GP prescriptions for COPD were higher in urban areas and northern regions of England, but less clear patterns were seen for asthma in comparisons using age-sex standardised event ratios. Holt et al. (2011) analysed hospitalisations within a Bayesian hierarchical framework, while other examples include Centers for Disease Control and Prevention (2008), Joo et al. (2007), Lipton and Banerjee (2006) and Nandram et al. (2000). Sofianopoulou et al. (2013) explored geographical patterns of GP drug prescriptions, considering the Newcastle and North Tyneside area in the UK as a study region. Studies (Hacking, Muller, Buchan, et al., 2011, Hansell, Hollowell, McNiece, Nichols, Strachan, 2003, Wells, Gordon, 2008) suggest that there is a significant difference of morbidity and mortality within and between regions of the UK over the last 40 years. This needs to be taken into account in order to both provide reliable statistical estimates, as well as to help public health policy makers more clearly identify target areas with great needs and improve disease prevention and treatment.

The objective of this study is to investigate trends in asthma and COPD at the population level across England by using multiple data sources to help understanding the relationships between disease prevalence, morbidity and mortality. We explore spatial and temporal patterns of GP drug prescriptions, hospital admissions and deaths, and we evaluate if different behaviours can be seen for different data sources which underline different condition severity. We also focus on the detection of unusual areas, i.e. characterised by a temporal trend which deviates from the general one, suggesting the presence of a policy or an emerged localised factor. In this analysis we combine information on asthma and COPD, given the similarities in these conditions, issues distinguishing between them (Drazen et al., 2015) and the fact that the GP prescription dataset used does not provide information on diagnosis.

The remainder of the paper is structured as follows. Section 2 describes the study design and the data sources used for the analysis, and Section 3 describes the statistical modelling framework. In Section 4 the results of the study are presented, followed by a discussion, and finally, Section 5 summarises the main findings of the paper, and suggests recommendations for future research.

## 2 Data sources

To gain a better understanding of asthma and COPD, we make use of three different data sources: (*i*) General practice (GP) drug prescription data of treatments used for these conditions, which capture patients with mild to severe symptoms and will give a general picture of the disease prevalence across the study region; (*ii*) Hospital Episode Statistics (HES) admissions with primary diagnosis of asthma or COPD; (*iii*) mortality data with asthma and COPD disease as cause of death. The latter two data sources will inform on cases characterised by higher severity. We are going to describe each data source in the rest of this section.

### 2.1 GP drug prescription data

The Prescription Cost Analysis (PACT) data are accessed from the NHS Business Services Authority. These include the monthly prescriptions of all drugs from 8003 general practices across England from August 2010 onwards at a monthly temporal resolution. In this study we use the prescriptions on Salbutamol, Ventolin and Clenil Modulite,with corresponding British National Formulary (BNF) codes 1011R0AAAPAP, 0301011R0BEAIAP and 0302000C0BPABBF respectively. These account for more than 90% of the total prescription of short acting beta2-agonist (SABA), a class of drugs that relieves patients from bronchospasm which characteristically occurs in acute symptoms (Drazen et al., 2015). Every GP is part of a local clinical commissioning group (CCG), which is the authority responsible for local healthcare services including local hospitals and NHS services, according to the 2012 Health and Social Care Act. GPs of the same CCG collaborate to evaluate local needs, monitor services, set priorities and make area-specific decisions to promote healthcare services for local residents. This suggests that GPs within the same CCG should share similarities. Therefore, the available GP data are aggregated at CCG (211 in England) level to be used for the analysis. The PACT data also contain the number of patients registered within each GP, with information on age group and sex. These are also aggregated at CCG level and they are used for the calculation of the expected number of drugs which will be the offset for the analysis of GP drug prescriptions.

### 2.2 HES and mortality data

Health data for England from August 2010 to March 2011 were obtained from the Small Area Health Statistics Unit (SAHSU) at Imperial College London. Hospital Episode Statistics (HES) admission data, supplied by the Health and Social Care Information Centre contain the number of admissions with a primary diagnosis of asthma or COPD, and may also include readmissions of the same patient. Mortality data were also obtained from SAHSU, supplied by the Office for National Statistics (ONS), derived from the national mortality registrations. The number of deaths with underlying cause of death (UCD) of asthma or COPD was collected. International Classification of Disease coding version 10 (ICD-10) was used for admission and mortality coding throughout this time period and asthma and COPD were defined as ICD-10 codes 490–496. Linkage between HES and mortality data to identify individual patients was not possible. Population data were also obtained from the Office for National Statistics, with individual-level information on age and sex and these were used for the calculation of the expected number of cases. HES, mortality, and population data were all aggregated to CCG level.

We have considered the same time period of August 2010 to March 2011 across all three datasets.

## 3 Statistical analysis

The analysis is conducted within a Bayesian hierarchical framework that takes into account the complex dependence patterns of asthma and COPD over space and time. Bayesian methods have been extensively applied in epidemiological studies, in order to summarise the spatial and temporal variations of the disease risk (Best et al., 2005). Approaches within the spatio-temporal setting have been suggested by many authors (Abellan, Richardson, Best, 2008, Bernardinelli, Clayton, Pascutto, Montomoli, Ghislandi, Songini, 1995, Knorr-Held, 2000, Knorr-Held, Besag, 1998, Waller, Carlin, Xia, Gelfand, 1997).

The spatio-temporal model that we use in this paper, known as BaySTDetect, is a recently developed method by Li et al. (2012) that is able to estimate spatial and temporal patterns, and to also detect areas whose temporal pattern deviates from the general one.

### 3.1 Model specification

The first level of the hierarchical model is given by

(1) $$\begin{array}{c} {\text{Y}}_{it}∼\text{Poisson}\left(\mu_{it}\;{\text{E}}_{i}\right) \end{array}$$

where Y_*it*_ and E_*i*_ are the observed and expected counts in CCG i=1,⋯,211 at time points t=1,⋯,8, corresponding to months August 2010, September 2010,..., March 2011.

In the second level of the hierarchy, the rate *μ_it_* follows a mixture of two components as follows:

(2) $$\begin{array}{c} \text{log}\left(\mu_{it}\right)=z_{i}\;\text{log}\left(\;\mu_{it}^{\text{C}}\;\right)+\left(1-z_{i}\right)\;\text{log}\left(\;\mu_{it}^{\text{AS}}\;\right) \end{array}$$

where

(3) $$\begin{array}{c} \text{log}\left(\;\mu_{it}^{\text{C}}\;\right)=\alpha_{0}+h_{i}+\gamma_{t}\;\;\;\;\text{(Common}\;\text{Model)} \end{array}$$

and

(4) $$\begin{array}{c} \text{log}\left(\;\mu_{it}^{\text{AS}}\;\right)=u_{i}+k_{it}\;\;\;\;\text{(Area-Specific}\;\text{Model)} \end{array}$$

The Common Model (3) consists of spatial and temporal effects, *h_i_* and *γ_t_* respectively, that are combined additively on the log scale, thus estimating the temporal pattern to be the same for all areas. An overall intercept *α*_0_ is also included.

To incorporate unusual temporal patterns that may occur in any particular month, we allow for the selection of an alternative Area-Specific Model (4), which estimates the temporal effects *k_it_* independently for each area. An area-specific intercept *u_i_* is also included in the model.

In the third level of the hierarchy, priors are specified for all model parameters as follows:

$$\begin{array}{ccc} & & \alpha_{0}∼\text{U}\left(-\infty ,+\infty \right)\;u_{i}∼\text{N}\left(0,1000\right)\\ & & h_{i}∼\text{N}\left(v_{i},\sigma_{h}^{2}\right)\;and\;v_{i}∼\text{ICAR}\left(\text{W},\sigma_{v}^{2}\right)\;k_{i,t}∼\text{ICAR}\left(\text{Q},\sigma_{ik}^{2}\right)\\ & & \gamma_{t}∼\text{ICAR}\left(\text{Q},\sigma_{\gamma }^{2}\right)\;\text{log}\left(\sigma_{ik}^{2}\right)∼\text{N}\left(\alpha ,\beta^{2}\right) \end{array}$$.

For the Common Model, a spatial convolution prior based on the standard Besag–York–Mollie formulation (Besag et al., 1991) is assigned to the spatial random effects. This combines a spatially structured component that follows a conditional autoregressive prior (ICAR) (Besag, 1974), accounting for spatial correlation in the data, and a spatially unstructured component following a Gaussian prior, accounting for heterogeneity in the data. For the spatial ICAR prior, we specify the neighbourhood structure by defining an adjacency matrix W of size N × N such that the diagonal entries *w*_*i, j*_ = 0 and the off-diagonal entries *w*_*i, j*_ = 1 if areas *i* and *j* share a common boundary, and 0 otherwise. The temporal effects are assigned the temporal analogue of the ICAR prior, thus accounting for the temporal correlation in the data. Similar to the spatial ICAR prior, the temporal neighbourhood structure is defined through a matrix Q, where $q_{h,t}=1$ if |h−t|=1 and $q_{h,t}=0$ otherwise, with *h* and *t* indexing units of time. For the Area-Specific Model, the same ICAR prior is assigned to the temporal component *k_it_*. In addition, a prior is assigned to the area-specific variances $\sigma_{ik}^{2}$ as an extra hierarchical level for identifiability reasons. For the overall intercept *α*_0_ and the area specific intercept *u_i_*, vague priors are specified.

A weakly informative half Normal prior N(0,1) is assigned to each of the parameters *σ_h_, σ_v_* and *σ_γ_* (Gelman, 2006), while a N(*α, β*^2^) prior is assigned to the $\text{log}(\sigma_{ik}^{2})$ with parameters *α* and *β*^2^ following priors N(0, 1000) and N(0, 2.5^2^), based on the specification by Li et al. (2012). As the latter prior is somewhat informative, sensitivity analysis is carried out to assess the robustness of the results.

The model indicator *z_i_* follows a Bernoulli (0.95) prior, selecting estimates from either the Common Model (z=1) or the Area-Specific Model (z=0). The parameter 0.95 reflects our expectations that only a small number of areas express an unusual temporal pattern.

To classify areas as unusual, we use the posterior estimates of the indicator *z_i_* representing how likely it is for area *i* to follow the Common Model, i.e. to exhibit a usual pattern in the risks, and we select the ones that satisfy the following condition: r_*j*_ ≤ 0.05, where r_*j*_ is the *j*th ordered posterior *z_i_*.

### 3.2 Applications

The model is fitted to all three datasets described in Section 2. In the prescription model, Y_*it*_ is the number of GP drug prescriptions in CCG *i* at month *t* and E_*i*_ are the expected counts. Appropriate covariates are also included in (2) to adjust for age and sex. As an indicator of age, we use the percentage of active population (age group 15 to 64), while as an indicator of sex, we use the male to female ratio.

In the admission model, Y_*it*_ are the observed cases of asthma and COPD admissions in CCG *i* at month *t* and E_*i*_ are the expected ones based on age and sex direct standardisation using the whole of England as standard population. Similarly, in the mortality model, Y_*it*_ is the observed number of deaths due to asthma or COPD in CCG *i* at month *t* and E_*i*_ is the corresponding expected number based on age and sex direct standardisation using the whole of England as standard population.

The models are implemented in OpenBUGS using Markov Chain Monte Carlo (MCMC) integration algorithms (Gilks, 2005). Three chains are run for each parameter per model with different initial values for 80,000 iterations, from which 20,000 are discarded as burn-in, and estimates are based on the remaining samples using only every 5th iteration to limit autocorrelation. The simulations took around 17 h per model on an Intel Xeon processor 2.50 GHz with 23.4 RAM. Convergence was assessed through trace plots, BGR statistic, and Monte Carlo error.

## 4 Results & discussion

### 4.1 Spatial patterns

**Fig. 1:**
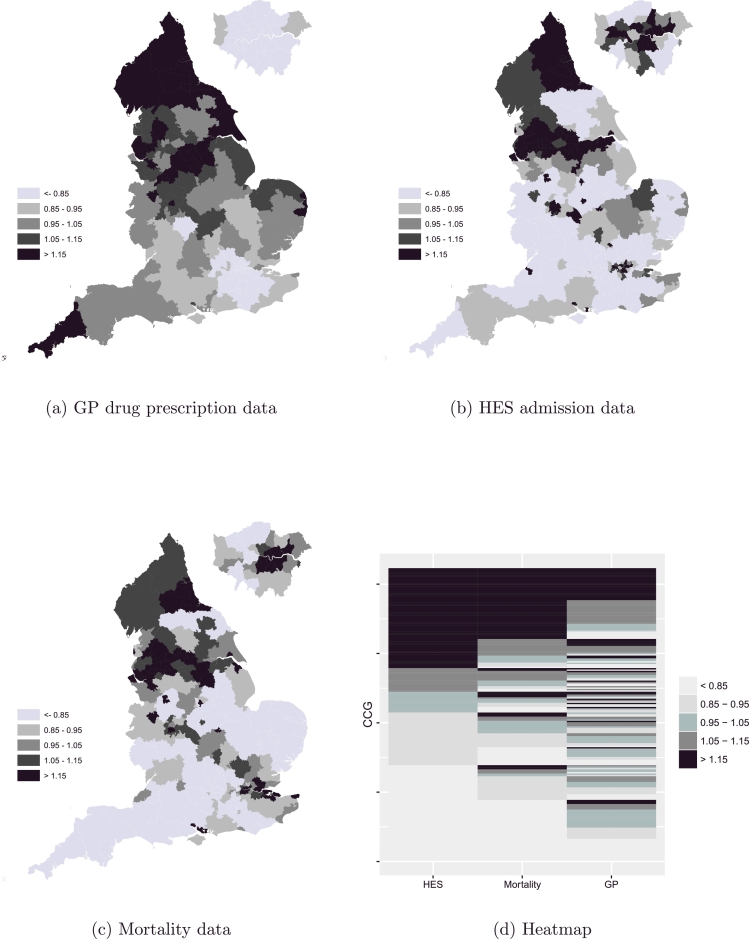
Spatial patterns of chronic respiratory disease across England for GP drugs (a), admissions (b), and deaths (c); heatmap showing correspondence across the three data sources (d).

We provide maps of posterior rates to investigate the geographical patterns of asthma and COPD across the three different data sources and evaluate whether these share any similarities or differences. Fig. 1a shows the residual relative risk of the spatial component exp(*h_i_*) of the Common Model (2) for the GP drug prescriptions across England during the period August 2010 to March 2011, while Fig. 1b and c show the corresponding risk for HES admissions, and deaths respectively.

A clear pattern is observed across all datasets with a strong increasing effect from south to north. High risk is focused mainly on the region that includes Liverpool, Manchester, Leeds, and Sheffield, as well as on the region Newcastle and Durham, with the highest risks being observed in the north east of England. Potential shared risk factors may include deprivation, which is highest in urban areas of the north-east and north-west, environmental factors such as air pollution and smoking prevalence (Andersen, Hvidberg, Jensen, Ketzel, Loft, Sørensen, Tjønneland, Overvad, Raaschou-Nielsen, 2011, Lopez, Shibuya, Rao, Mathers, Hansell, Held, Schmid, Buist, 2006, Prescott, Vestbo, 1999). The north-west is the areas with the highest prevalence of smokers (HSCIC, cited October 2015).

By comparing the risk distribution across different data sources, we observe that for admissions and deaths, these are somewhat consistent (Fig. 1b and c), while for prescriptions important differences are highlighted (Fig. 1a). This observation is confirmed by the heatmap in Fig. 1d which shows the correspondence across the three data sources using the spatial relative risk; the area in light grey represents the CCGs in England that share a low relative risk while the area in dark grey represents the ones with a high relative risk. It is clear that the proportion of areas with a low/high relative risk across all three data sources is relatively small compared to the proportion of areas with a low/high relative risk among HES admissions and mortality data. The correlation of the latter two was estimated to be 0.77, while the corresponding correlations of GP drugs and mortality, and GP drugs and HES admissions were close to 0.5.

Low risk is observed for admissions and deaths (Fig. 1b and c) in the south of England, and in the coastal areas. At the same time these areas exhibit high prescription rates (Fig. 1a). This suggests a number of hypotheses for further investigation e.g. that higher prescription rates are a causal factor in lower morbidity and mortality, that there are more patients with milder disease in the south-east potentially related to lifestyle factors (lower smoking) and environmental factors (lower air pollution), that deprivation results in higher morbidity and mortality but lower use of primary care. Age-sex effects could also explain these differences; these are accounted for at an aggregate level for drugs, through the inclusion of appropriate covariates at the GP practice level in the model, since information was not available at an individual level, while direct standardisation was used for admissions and mortality.

Only a few CCGs in the south appear to have high admission and mortality rates (Fig. 1b and c), including Portsmouth and Southampton CCGs. Increased smoking prevalence and high deprivation can be related to this. Unlike the rest of big cities in England, London appears to have low prescription rates (Fig. 1a) potentially related to the low number of smokers in the area (HSCIC, cited October 2015). Comparing between mortality and admission spatial patterns, a stronger south to north effect can be seen for mortality (Fig. 1c), showing the south eastern and central west parts of England as the least risky for disease death. Another region which potentially represents a group of patients with mild symptoms is around the Yorkshire area, where national parks are, and this area clearly stands out in the admissions map (Fig. 1b).

### 4.2 Temporal patterns

**Fig. 2:**
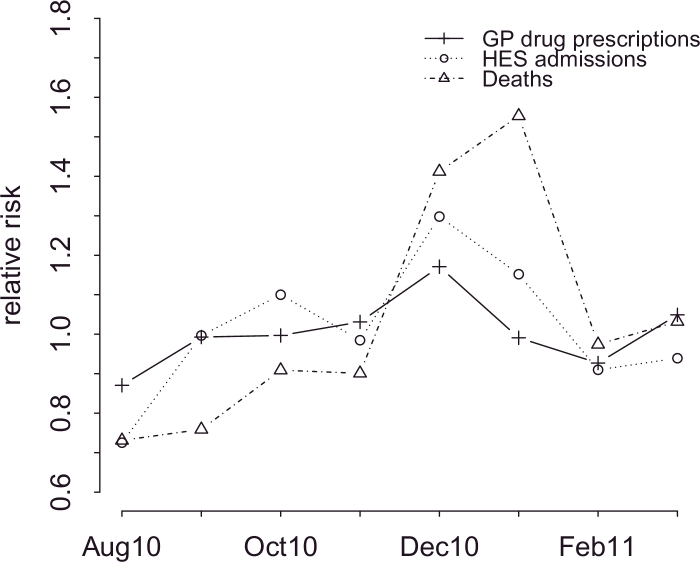
Temporal trends in chronic respiratory disease across different data sources.

The general temporal patterns in England from August 2010 to March 2011 under the different datasets considered in the study can be seen in Fig. 2, which plots the component exp(*γ_t_*) of the Common Model (2).

Generally, there was a seasonal pattern in the monthly risk for asthma and COPD across all data sources over the period August 2010 to March 2011 however the trends show important discrepancies. The highest risk was recorded around Christmas across all data sources. This is likely to represent peaks in respiratory infections, especially influenza and respiratory syncytial virus (RSV) that peak around this year. These result in increased respiratory symptoms from mild to severe and cause exacerbations of disease that are occasionally fatal (Fleming et al., 2015). The lowest peak was recorded in August for all three data sources, a period when people are on holidays, away from their homes. The raise in the rates across all datasets around September coincides with the start of term at schools.

By comparing temporal trends across different data sources, we observe that there is a high temporal variation for mortality, whereas this is lower for admissions and prescriptions. Interestingly, a time lag is apparent between admissions and mortality. The highest peak for admissions is December, whereas for mortality is January and this might reflect the group of admissions which were then followed by death.

### 4.3 Detection of unusual areas

Finally, we obtain the posterior estimates of the indicator *z_i_* for each model and we classify areas as unusual based on the rule described in Section 3.1.

**Fig. 3:**
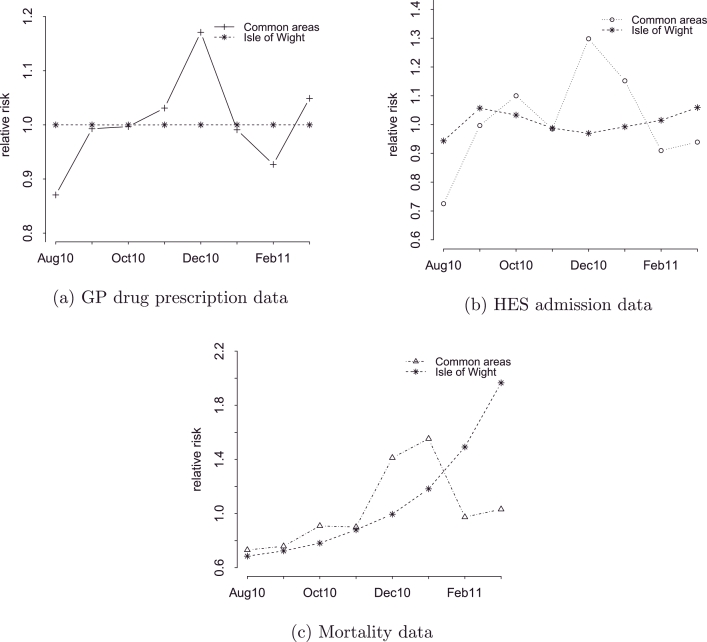
Temporal trend across different data sources for Isle of Wight.

For GP drug prescription and mortality data, no areas were detected as unusual, meaning that all posterior estimates of the parameter *z_i_* were above our *a priori* threshold value of 0.05. When ranking areas by probability, the Isle of Wight was the area with the smallest probability of following either the common prescriptions temporal pattern (with a probability of 0.13), or the common mortality temporal pattern (with a probability of 0.11). Looking at the temporal patterns for this area in Fig. 3, we see that for prescriptions, a flat pattern is apparent, indicating low rates with no peaks throughout the whole time period (Fig. 3a), while for mortality, a near exponential increase is observed, which after December 2010 exceeds the national death rates importantly (Fig. 3c). The corresponding plot for the admissions shows a stable pattern (Fig. 3b), similar to the one for prescriptions (Fig. 3a).

After investigating the CCG of Isle of Wight, we found that an intervention was implemented in order to reduce the high prevalence of long respiratory diseases in the area, which was combined with an excess expenditure on respiratory medication. The project entitled ‘Isle of Wight Respiratory Inhaler Project’, led by the National Institute for health and Clinical Excellence (NICE), involved training of healthcare professionals in the use of the inhaler, patient training, and assessment of the inhaler technique. According to the results reported in 2009, the costs on selective beta-agonists fell by 22.7%, the number of prescriptions fell by 25.2%, the emergency admissions due to asthma were reduced by 50%, and associated deaths by 75% (NICE, , 2011).

Given the above, the flat pattern in prescriptions seen in Fig. 3a is well supported, as well as the one for admissions (Fig. 3b). However, the increasing temporal trend in deaths in Fig. 3c which peaks in the last month of the study period generates questions. Since the findings reported by NICE consider only asthmatic patients, meaning that COPD patients that use the same medication are not represented, further analysis is required for the CCG of Isle of Wight separately on asthma and COPD in order to understand the underlying causes of the mortality trend. Additional data need to be also analysed in order to see the progression of the rates long after the intervention was implemented. Moreover, COPD is often misdiagnosed as asthma due to the similar symptoms that these two diseases share, and also depending on the availability of spirometry (Walker et al., 2006). This calls for further investigation into the misdiagnosed cases of COPD as asthma in the CCG of Isle of Wight which might be an adverse effect of the project.

**Fig. 4:**
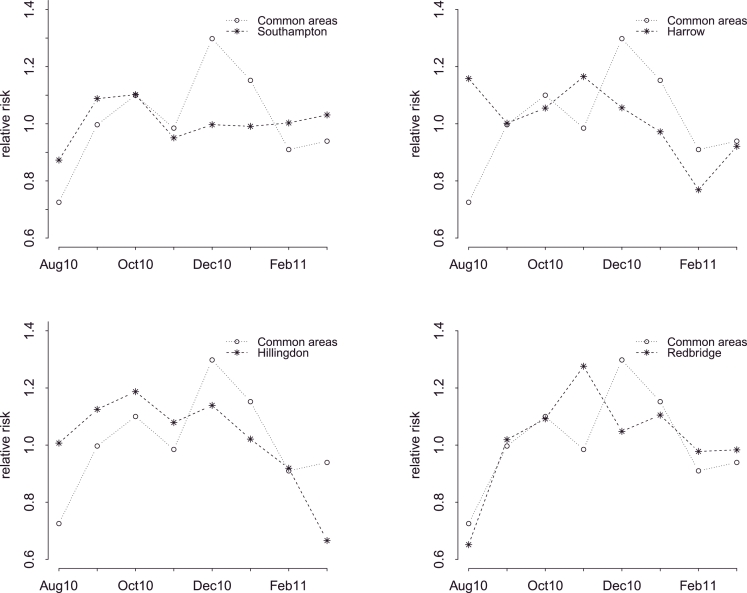
Unusual temporal trends under HES admissions data.

On the other hand, four areas were detected as unusual using a 0.05 threshold level for hospital admissions model: Harrow, Hillingdon, Redbridge and Southampton. The time plots in Fig. 4 show the temporal trends for each unusual area, compared to the general temporal trend.

As it can be seen, the corresponding areas do not follow particularly extreme patterns, but there are discrepancies from the common one that the model identifies as important. For instance, although Harrow in general exhibits lower than the average national admission rates due to asthma or COPD, an unusual high peak is observed in August, as well as one in November, which months are considered safe for the rest of England. Hillingdon, on the other hand appears to have an extreme decrease in March 2011. Redbridge shows a different pattern in prescriptions from the other areas in that prescriptions increase in early November. London is a city known for its diversity, and for the population movements within it, hence it is not easy to make conclusions as to what has happened in those CCGs. Factors such as differences in local population characteristics, or differences in the quality of hospital care and the support people receive to manage their condition across time could be responsible.

The fourth area that was detected as unusual in terms of admission trends is Southampton. This is one of the few areas on the South Coast that show an increased risk for severe symptoms and deaths due to asthma and COPD (Fig. 1). Interestingly, it seems that the low risk observed in Fig. 4 from December onwards is the outcome of an intervention that was implemented in order to address the excess number of admissions in the CCG of Southampton. Wilkinson et al. (2014) found that 34 patients were responsible for 22% of the total COPD admissions over a 3-year period. The authors present an admission avoidance strategy that was constructed for this group of patients, and as a result the readmission rate fell from 13.4 to 1.9%.

## 5 Conclusions & further recommendations

In this paper we have investigated the spatial and temporal patterns of asthma and COPD, with a special focus on the detection of areas that follow an unusual temporal pattern compared to the general one, by using effective high quality datasets, aggregated by CCG which is meaningful for healthcare practices. There are two main conclusions we have drawn from our study.

First, we have shown that multiple data sources representing different degrees of disease severity give a more comprehensive picture of the respiratory health problem with potential implications for healthcare. Some similarities across the different datasets both in the spatial and temporal variation are indicated, however also important discrepancies are apparent reflecting the different groups of patients that are represented.

Second, the detection mechanism that is provided by the model we used, together with inference on the spatial, and temporal variation, can aid health care professionals and public health practitioners identify target areas, assess a policy impact or the quality provided by hospitals, and hence develop effective prevention programs to improve population health.

A strong aspect of the modelling approach is that it identifies unusual behaviour not only in terms of increased risk, but also in terms of any risk pattern that deviates from the expected one. Additionally, it only detects areas when a certain criterion is met, and the specification of this depends on how conservative we want to be. In the original paper the model was applied on annual data by Li et al. (2012), while here we used monthly data as we were interested in the seasonal pattern of asthma and COPD. A great advantage of the additive model specification is that it allows for such flexibility, through the temporal random effects which account for unmeasured covariates that vary across time. Li et al. (2012) chose a Bayesian False Discovery Rate (FDR) to adjust for multiple testing, following Newton et al. (2004). Alternative FDR approaches have been suggested by other authors (Catelan, Lagazio, Biggeri, 2010, Muller, Parmigiani, Rice, 2006, Storey, 2003, Whittemore, 2007). Here, we chose to adopt a standard classification rule, as we believe that the above FDR rule does not apply in our case, given the small number of positives we have. Besides, it is argued whether there is a need to perform multiple testing correction within a Bayesian framework (Gelman et al., 2012).

Asthma and COPD are jointly considered in the analysis, as the indication of the disease was not possible for the prescriptions, and one of our objectives was to compare between the different data sets. A next step is to separately explore asthma and COPD trends for all ages and for children, adults and elderly to see if our findings hold for these conditions individually, and to better understand the temporal patterns of the areas found to be unusual. Further analysis should formally investigate possible confounders, effect modifiers and causal factors such as deprivation, smoking, seasonal respiratory virus activity and service access, and to also adjust for socio-economic status, as this is likely to affect the spatial patterns. It is relatively easy to introduce covariates into the BaySTDetect model to start to explore some of the hypotheses as to why some CCGs differ from national trends. As an example we introduced a variable to represent deprivation — the Index of Multiple Deprivation (IMD) score, which was initially available at LSOA level for England. This was aggregated at CCG level, and then converted into a categorical variable of 5 levels (quintiles) which was introduced into the model through dummy variables. The results showed no important effect; the exponential of the posterior estimates of the coefficients of the dummy variables varied from 0.99 to 1.02 with all credible intervals including 1. The inclusion of deprivation in the model did not affect the posterior estimates of the other parameters, nor the areas that were detected as unusual.

In addition, a multivariate modelling approach will be considered, which could provide a more comprehensive picture of the trends and potential outbreaks of the disease, by borrowing information across different data sources. Finally, it would be useful to know the exact temporal point at which an unusual observation occurs, and the model should be modified to adjust for this.
